# Navigating the Rapids: The Development of Regulated Next-Generation Sequencing-Based Clinical Trial Assays and Companion Diagnostics

**DOI:** 10.3389/fonc.2014.00078

**Published:** 2014-04-17

**Authors:** Saumya Pant, Russell Weiner, Matthew J. Marton

**Affiliations:** ^1^Merck Research Laboratories, Molecular Biomarkers and Diagnostics, Rahway, NJ, USA

**Keywords:** companion diagnostics, disruptive technology, precision medicine, next-generation sequencing, clinical next-generation sequencing, molecular diagnostics, drug development strategy, mutation detection methods

## Abstract

Over the past decade, next-generation sequencing (NGS) technology has experienced meteoric growth in the aspects of platform, technology, and supporting bioinformatics development allowing its widespread and rapid uptake in research settings. More recently, NGS-based genomic data have been exploited to better understand disease development and patient characteristics that influence response to a given therapeutic intervention. Cancer, as a disease characterized by and driven by the tumor genetic landscape, is particularly amenable to NGS-based diagnostic (Dx) approaches. NGS-based technologies are particularly well suited to studying cancer disease development, progression and emergence of resistance, all key factors in the development of next-generation cancer Dxs. Yet, to achieve the promise of NGS-based patient treatment, drug developers will need to overcome a number of operational, technical, regulatory, and strategic challenges. Here, we provide a succinct overview of the state of the clinical NGS field in terms of the available clinically targeted platforms and sequencing technologies. We discuss the various operational and practical aspects of clinical NGS testing that will facilitate or limit the uptake of such assays in routine clinical care. We examine the current strategies for analytical validation and Food and Drug Administration (FDA)-approval of NGS-based assays and ongoing efforts to standardize clinical NGS and build quality control standards for the same. The rapidly evolving companion diagnostic (CDx) landscape for NGS-based assays will be reviewed, highlighting the key areas of concern and suggesting strategies to mitigate risk. The review will conclude with a series of strategic questions that face drug developers and a discussion of the likely future course of NGS-based CDx development efforts.

## Introduction

The concept of personalized medicine relies heavily on access to information on an individual’s unique genetic characteristics to tailor therapy. However, the current paradigm of regulated molecular diagnostic (Dx) testing, in which individual Food and Drug Administration (FDA)-cleared Dx tests are employed to detect mutations in a single gene, sits uneasily in this framework of personalized medicine (1, 2). The advent of clinical next-generation sequencing (NGS) has begun to provide to the clinic a more expansive insight into genetic mutations in a broader set of genes, usually drawn from pathways implicated in and actionable by current therapeutics or by promising drug candidates in development (3). NGS-based diagnosis is specially promising for diseases that have a highly complex and heterogeneous genetic composition. The field of oncology is therefore very well positioned to benefit greatly from such an approach (4, 5). Since NGS-based technology permits a more complete view into a tumor’s genetic composition, it is easy to foresee that treatment paradigms must change accordingly to allow treatment based on the molecular pathological fingerprint of the individual. As a result, the question is not technological (“Can it be done?”), but rather practical (“How can NGS technology be developed into a mainstream multi-gene or multi-transcript Dx fingerprint?”) and regulative (“What are the barriers that must be overcome for this disruptive technology be approved as a general companion diagnostic (CDx) device for multiple therapeutics?”). It is clear the scientific community is rapidly embracing the technology as NGS-based tests are being employed across multiple disease areas, including oncological, metabolic, cardiovascular and neurosensory disorders, and in prenatal diagnoses (6–10) where genetic components are defined. As of late 2013, several dozen clinical labs offer over 50 different laboratory-developed tests (LDTs) using NGS (11). These tests are offered as single-gene assays or multi-gene or multi-transcript panels. Commercially available NGS-based cancer panels are already being used in clinical practice and as clinical trial assays (CTAs) to guide patients to most appropriate experimental treatment (8, 12, 13). Nonetheless, there are no FDA-approved NGS CDxs available today and there are significant challenges in developing such tests. We compare developing NGS-based Dx to navigating the rapids, an exercise full of challenges, continuously changing technologies, policies, and regulations as the field develops at a rapid pace, and yet the promise of personalized medicine is within reach and closer than ever before.

## Current Paradigm is Unsustainable

Precision medicine has been defined as identifying the right drug, for the right patient, at the right dose, at the right time (14). Intrinsic to identifying the right patient is a Dx device. If it is linked to a specific therapeutic and if the test is required for the safe and effective use of the drug, then Dx device is termed a CDx. The current testing paradigm for precision medicine links a specific drug to the Dx (15, 16) and can be summarized as “one-drug/one-gene Dx.” This is abundantly illustrated for FDA-approved Dxs, such as the one-gene tests approved for mutations in *EGFR*, *KRAS*, and *BRAF*. Yet, it is equally clear that the current paradigm is not sustainable (17, 18). First, cancer is an exceedingly complex molecular and epigenomic disorder, resulting from perhaps hundreds of different molecular defects, including somatic mutations, gene expression changes, and genome rearrangements. Furthermore, tumorigenesis and tumor progression are driven by altered gene regulation networks that are not always tractable to a clear and defined somatic mutation (19). Recent results from clinical studies support the emerging concept of the “mutation signature” or spectrum of correlated mutations in cancer (20, 21), which postulates that the combination of mutations present is more predictive of the response to treatment than individual gene mutation status. Thus, to ensure their patients are offered the best possible treatment, physicians will want to examine the tumor’s whole cancer genome, both somatic mutation and transcriptional changes, to identify the most personalized therapy, and they will do so whether or not there is a FDA-sanctioned Dx for a particular drug. Instead, they will use LDTs, which the FDA believes should be regulated as *in vitro* diagnostics (IVDs) (75 FR 34463, 2010). Thus, the current situation is untenable since it is only a matter of time before more comprehensive tests will routinely be used to diagnose a patient’s tumor. Second, not only do physicians need more molecular information, but patients want it too. In this age of internet medicine, many patients are well-informed and strongly advocate for more comprehensive testing, even to the point of paying for it themselves in order to get a more complete picture of their cancer (22). Their reasoning that more information is better is hard to argue against. Hospitals and for-profit companies have developed tests to meet this need, and advertisements for comprehensive genomic tumor assessment on television, radio, and internet are not uncommon. Furthermore, patients considering a clinical trial at a major hospital are beginning to expect molecular characterization of their tumor as a *quid pro quo* for participation in the clinical study. A third, more practical, reason why the current model is not sustainable is the limitation of tissue. A Dx tumor specimen block can only be sectioned into a limited number of sections. Sample is limiting and tests are not currently multiplexed; separate slides are usually required for different immunohistochemical (IHC)-, ribonucleic acid (RNA)-, or deoxyribonucleic acid (DNA)-based tests. In most cases, there is simply not enough material to test for every gene mutation that is available, and therefore a more efficient use of the patient’s specimen is needed. For these reasons, it is clear the “one-drug/one-gene Dx” paradigm is unsustainable and that the drive toward precision medicine is changing clinical practice, and as it does, it will change the clinical testing paradigm for cancer treatment decisions.

## Disruptive Shift

Next-generation sequencing is a classic disruptive technology (23). It may even change the way precision medicines are developed (24). Although these changes will impact the healthcare community and their patients, in this section we will only focus on the potential impact on drug developers and manufacturers of Dx tests. The crux of these changes is the shift from a “one-drug/one-gene Dx” model to a “multi-gene Dx/many drugs” paradigm (25, 26). An oversimplification of the interaction between the drug developer and the Dx company can be summarized as: the drug company develops a promising drug and discovers late in development that a Dx is needed to identify the appropriate patient population. Then it works with the Dx company to develop the test to detect and/or quantify the specific biomarker, and they are both tested in pivotal trials. Thus, the drug drives the device development. The use of a multi-gene or multi-transcript panel has the potential to change that. Instead of a single drug developer partnering to develop a single Dx test, what may happen is that the device manufacturer may design an assay able to detect a myriad of RNA or DNA biomarkers. That is, the device manufacturer may drive content on the device and may proactively seek FDA-clearance independent of a partnership with a drug maker. The implication of this disruptive shift is a set of challenges that will be discussed in a later section.

## Primer on NGS Platforms

Several firms have developed small benchtop NGS sequencers for the clinical Dxs market. The current leading platforms are the MiSeq from Illumina, Inc. and the Personal Genome Machine (PGM) from Life Technologies, Inc., which together comprise >85% of market as of early 2014 (Bloomberg Businessweek, January 2014). The recent agreement between Roche Molecular Diagnostics and Pacific Biosciences (PacBio) heralds the entry of the latter into the Dx arena. Qiagen has announced that it will release its benchtop GeneReader™ NGS platform in 2014. Key factors that influence clinical labs’ adoption of a particular platform include sequencing quality, turnaround time (TAT), cost per sample, optimal ease of use for the operator, and sample multiplexing capability (recognizing that multiplexing is likely required to reduce cost). We provide a brief overview of the main clinical NGS technology platforms here and refer the reader to exhaustive reviews on NGS technology and instrumentation advances for further details on each (27–30).

### Ion Torrent

Life Technologies’ Ion Torrent semiconductor sequencing technology, which made its debut in 2011, is based on a sequencing-by-synthesis approach in which individual templated DNA molecules positioned in microwells on a semiconductor chip are sequentially incubated with each of the four deoxynucleotide triphosphates (dNTPs) to support DNA strand polymerization (31). Only the dNTP complementary to the template is incorporated at the end of each template strand. As each dNTP is incorporated, a proton is released, which acts as an indicator of base incorporation and the number of bases incorporated consecutively. The resulting pH changes are recorded as voltage changes that convey the sequence of bases for the flow. Advantages of this technology include optics-free readout, low input DNA requirement (which is critical for clinical practice), and longer read length with accurate base calling (32).

### Illumina

The Illumina technology also utilizes a sequencing-by-synthesis approach with bridge amplification (27). Clonally amplified DNA templates are immobilized to an acrylamide coating on the surface of a glass flowcell that serves as the reaction and sequencing substrate. Fluorescently labeled reversible-terminator dideoxynucleotide triphosphates (ddNTPs) are added one base at time in this sequencing technology. After the addition of each nucleotide, the clusters in the flowcell are imaged to determine which fluorescent dye was incorporated. In its current manifestation, Illumina’s greatest strength is the easier workflow of the amplicon library preparation and reduced hands-on time as compared to other platforms. Data from research versions of the technology, such as the larger HiSeq platform, associates Illumina with greater accuracy of base calls and lower indel detection errors (29).

### Pacific Biosciences

To compete in the clinical and Dx space, PacBio introduced the desktop RS machine in 2011. PacBio utilizes single molecule real time (SMRT) sequencing. DNA template bound to DNA polymerase molecules is attached to the bottom of 50 nm-width wells termed zero-mode waveguides (ZMWs). Each polymerase molecule carries out second strand DNA synthesis using γ-phosphate fluorescently labeled nucleotides present in the reaction mix. The ZMW width does not allow light to propagate, but energy penetration excites the nucleotide fluorophores in the vicinity of the polymerase at the bottom of the well. As DNA synthesis occurs, the incorporation of each base creates a distinctive pulse of fluorescence, which is detected and recorded in real time (33). In a platform comparison of the three technologies, Quail et al. noted the high fidelity of PacBio data and the ability to read long sequences (28), but added the caveat that very high read depth is required for achieving accuracy near that of MiSeq and PGM. Additionally, in the context of formalin fixed paraffin embedded (FFPE) and fragmented DNA material, PacBio’s long read strength may not be of great advantage.

It must be noted that the rapid pace of performance improvement of both the Illumina and Life Technologies benchtop sequencers has been instrumental in making NGS-based Dxs within reach (34). Both platforms have incrementally increased the quantity and quality of base calling while reducing library preparation time and allowing on-instrument primary and secondary data analysis, which was considered the largest bottleneck to clinical and Dx NGS up to early 2011. For example, advances in library preparation have reduced processing times two-fold compared to older version kits available from both companies in 2011. On the instrumentation side, the new, smaller instruments (MiSeq and PGM), have enhanced output and accuracy of base calling compared to the earlier larger throughput NGS instruments (Illumina GAIIx, Illumina HiSeq 2000, and earlier versions of PGM) (28). An Ion Torrent 318 chip with 400 bp sequencing reads can easily produce >1 Gbp aligned data passing Q20 scores. Furthermore, the newer versions of chemistry have significantly improved the average error rates over the length of reads. Also, the design of the new emulsion PCR (ePCR) Ion One Touch 2 system released in late 2012 increased the uniformity of sequencing by enhancing inclusion of low length template Ion Sphere particles (ISPs) in the template and enhancing library templating for sequencing. Additionally, on-instrument analysis improvements significantly reduced the challenges and time constraints imposed by bioinformatic analysis. Although even more improvements are anticipated, these technical advances have made clinical NGS a reality.

## Operational Challenges for NGS Assays in the Clinic

### Specimen type and amount

One of the key considerations with current clinical NGS tests with Dx aspirations is the reliance on FFPE material. DNA isolated from FFPE specimens presents unique challenges in being highly degraded and of poor quality compared to that from fresh frozen specimens (35). This places a limitation on the size of amplicons that can be reliably amplified from this material, with tests targeting amplicon targeted regions from around 120–180 bp (Ion Torrent AmpliSeq Cancer Hot Spot panel)^1^ to ~175 bp (Illumina TruSeq and TruSight assays)^2^. Additionally, DNA derived from FFPE material undergoes cytosine deamination during the fixation process, which can complicate analyses in downstream Dx applications unless a downstream bioinformatic solution is able to address and compensate for such base alterations (36, 37). What is perhaps an equally great challenge is the amount of specimen required for the assay. Ion Torrent assays for cancer mutational hot spot panels require about 10 ng input of FFPE DNA, the Illumina TruSight clinical assay panel requires 30–300 ng input DNA (as determined by quantitative polymerase chain reaction (qPCR)-based functional DNA assessment) and a majority of the established clinical NGS panels available as lab-developed tests require about 40 μm FFPE material or >100 ng input DNA, in addition to sections for pathology review and tumor markup. In contrast, individual Dx tests using either traditional Sanger sequencing or other PCR-based assays typically require at least 15 μm input per assay. This apparent drawback of large input NGS-based testing (particularly for Illumina assays) has led to methods to reduce sample requirements, such as Rubicon Genomics ThruPLEX kit, Illumina’s Epistem technology, NuGen amplification products, and New England Biolabs NEBNext Ultra for low input NGS. Importantly, the assay manufacturers have themselves adopted steps to further decrease input amount for assays without compromising on test sensitivity. One final note: for NGS-based tests, the sample requirement for material is relatively independent of the number of genes in the assay since the test requires the input of a minimal number of amplifiable genomes only (38).

### Assay turn-around time

A major hurdle in the adoption of a NGS-based test as a CTA is the logistics in terms of the length of time from sample collection to reporting of results. Most clinically applicable NGS-based tests require 7–14 business days TAT (39). In the case of hematological malignancies, such a long reporting time seems to be clinically untenable. Some clinicians are hesitant to use NGS tests for patient stratification and prospective enrollment in trials because patients may not be willing or able to wait 2 weeks for a test result, and thus will pursue other clinical trials in the meantime. As the NGS assay TAT continues to improve (discussed under analytical challenges) this is likely to be a smaller concern in the next few years.

### Availability of CROs with CLIA NGS capabilities

Clinical trial sponsors typically prefer to perform clinical trial sample analysis in a single central lab to avoid potential liabilities of using multiple local hospital laboratories, which can compromise results or complicate interpretation due to the use of different tests, different instruments, different validation standards, and quality control (QC) processes, and different histopathological practices such as macrodissection (14, 40). Unfortunately, despite the potential commercial opportunity that available NGS-based multi-gene panels represent, only a few contract research organizations (CROs) or specialty testing labs have invested the effort to develop the expertise to offer NGS services as Clinical Lab Improvement Amendment (CLIA) laboratory tests suitable as CTAs. Thus, the majority of the technical expertise does not reside in traditional central labs and CROs (11), but rather in academic institutions and in large clinical hospitals, where medical practitioners have begun to use NGS-based mutational profiling screening to match their patients to the appropriate therapeutic (41). These factors represent a significant challenge for pharmaceutical companies interested in developing NGS-based CDxs.

The concern about using local laboratory for enrollment to clinical trials comes from several different areas. First, there may be variability due to different interpretation of the various guidelines, checklists, and recommendations available for NGS assays (42–44) since laboratory directors have some discretion and may interpret the rules differently in some cases. An example is the interpretation of the College of American Pathologists (CAP) NGS checklist that recommends orthogonal analytical confirmation of all encountered mutations from an assay before the mutation is reported as clinically actionable (43). This guidance seems to be interpreted differently in different labs based on the availability of subjects, which limits the probability of encountering samples with said mutations. Second, the current lack of standardization between hospital laboratories, especially in analytical and post-analytical processes, introduces risk in, for example, mutation calls for the same samples since they may utilize different platforms, assays, software, and algorithms to make mutation calls. This is even seen for simpler, non-NGS-based assays such as for *KRAS* mutation detection assays. In a retrospective study (29) in which specimens from colorectal cancer patients treated with panitumumab (an anti-epidermal growth factor receptor gene (EGFR) monoclonal antibody) were analyzed for the presence of activating *KRAS* mutations in both local hospital labs and a centralized testing facility at a CRO, the authors found that 6 of the 60 patients tested had mutations and should have been excluded from the study. The conclusion was that the LDTs in local hospital labs failed to detect the *KRAS* mutations, allowing ineligible patients to be enrolled, and thereby diluted the drug response rate since patients with *KRAS* mutations were not expected to respond to panitumumab treatment (45). That this can happen with a simple PCR-based mutation test illustrates the risk associated with complex assays such as NGS-based assays (43).

The challenge for the pharmaceutical company is how to run a clinical trial that maintains the homogeneity of the trial population in light of the paucity of CROs with CLIA NGS capabilities. Some have suggested to use the local lab test as a CTA for enrollment but confirm the result with a centralized assay or to use the local lab test as a screen to identify patients whose samples should be analyzed by the centralized CTA. Both of these suggestions are problematic. First, analyzing the patient specimen by two assays unnecessarily consumes limiting material. Second, discordant calls are inevitable, especially for assays as complex as NGS-based assays. Determining which of two discordant results is accurate will likely be time-consuming and expensive. Furthermore, the discordant data will likely raise concerns of any regulatory agency reviewing the clinical trial and it may call into question the accuracy of the CTA. Similarly, the idea to use local lab assays to screen patients for subsequent central lab testing will definitely introduce a patient population bias if the study only enrolls biomarker positive patients (12), and it may introduce a bias even if the study has both biomarker positive and negative arms. In general, it seems better to focus on reducing the TAT of sample analysis at the centralized laboratory than to rely on local laboratories for patient eligibility decisions.

A new paradigm in clinical NGS testing is the emergence of companies like Foundation Medicine (FM) and Personal Genome Diagnostics (PGD), which offer NGS-based panel tests as CTAs to support clinical trials as well as directly to physicians. Boston-based FM offers the Foundation One panel that reports on the mutational status of 285 genes that are found to be commonly mutated in cancers; it has also recently announced a similar panel for hematological malignancies (46). PGD, based out of Baltimore, offers a clinical targeted cancer gene panel cancer select for the detection of genetic alterations in 120 well-characterized cancer and pharmacogenomics genes (47). These companies thus offer an alternative to local laboratory testing for clinical trials. Companies can either use one of these commercial panels as a CTA or can establish a clinical trial protocol that enables recruitment of subjects that have already had the tests performed (13, 48, 49).

### FDA-cleared instrumentation

Although Illumina’s MiSeqDx instrument received CE marking in June 2013, the lack of commercially available instrumentation was a major hurdle to CDx development prior to the FDA-clearance of Illumina’s MiSeqDx platform as a class II device in November 2013 [510(k) number K123989]. In addition, the FDA also made the device and substantially similar devices exempt from the premarket notification requirements. At the same time, the FDA-cleared Illumina’s cystic fibrosis carrier screening assay, an assay that detects all 139 variants in the cystic fibrosis transmembrane conductance regulator (CFTR) gene, as well as an assay for CF diagnosis by sequencing all the medically relevant regions of the CFTR gene assay (Source accessdata.fda.gov and illumina.com). The type of data required for these submissions provides the first documented and public view into the Center for Devices and Radiological Health’s (CDRH) specific expectations for verification and validation of NGS-based Dx tests; see below for a section in which this is discussed in detail.

Life Technologies’ has recently stated that its Ion Torrent PGM Dx System will be registered as a class II 510(k)-exempt device with the FDA, as opposed to applying for 510(k) clearance as was done for the Illumina MiSeqDx (50). This is apparently prompted by the FDA decision that the MiSeqDx instrument and substantially equivalent devices of that generic type will be classified into class II and be exempt from premarket notification requirements [510(k) K123989]. The Ion Torrent PGM Dx will be building on Life Technologies’ expertise with Dx instruments such as the 510(k)-cleared 3500 Dx Genetic Analyzer. The PGM Dx instrument will be an open platform for NGS tests but without specific assays submitted to the FDA. Life Technologies has stated that Dxs manufacturers applying for tests on the PGM Dx will reference the master file as needed to support their submission to the FDA and those assays would be evaluated by the FDA through either the 510(k) or pre-market approval (PMA) processes. The Ion Torrent system has one significant difference in that it includes two peripheral accessory instruments, the Ion OneTouch Dx for ePCR-based template preparation and the OneTouch ES Dx for magnetic bead-based ePCR library enrichment.

Pacific Biosciences RS II DNA Sequencing System’s regulatory path is currently not clear. However, in a significant move recently, Roche Diagnostics and PacBio entered into an agreement to develop Dx sequencing systems and consumables utilizing PacBio’s SMRT technology. Per this agreement Roche will become the exclusive worldwide distributor for PacBio’s human IVD products (51).

## Technical and Analytical Challenges for NGS Assays in the Clinic

### Design of the NGS assay

The first challenge toward a successful NGS CDx is the assay design. Most current clinical NGS assays rely on a hybrid-capture or PCR amplicon-based approach to provide overlapping, high density coverage across regions of interest (52). When working with FFPE biopsy specimens, the number of amplicons needs to be judiciously optimized to allow efficient coverage of large regions while keeping amplicon size small to enable efficient amplification of formalin-damaged DNA (53). The choice of platform and the degree to which the assay needs to include promoter, 3′ untranslated region (UTR), splice sites, or introns also affects assay design. Currently, most commercially available panels only cover exonic regions. While Ion Torrent’s hot spot mutation panels cover shorter fragment amplicons, Illumina’s exon coverage-based design tends to favor longer amplicons. While overlapping longer amplicons may increase the fidelity of readout by utilizing multiple overlapping fragments per base, amplicon length must be judiciously balanced to enable FFPE fragmented DNA analysis.

Genomic complexity of the region of interest can impact accuracy and precision of an assay (54), so it is also important to understand and to give due consideration to the same in assay design. Since the genome has been shown to replicate at different times with variable error as a function of time of replication, the analytical parameters including error rate must be calculated accordingly for specific regions based on sequence context (55, 56). Knowing whether the region of interest is a region of lower intrinsic fidelity allows one to improve accuracy by compensating with higher read depth. Similarly, the degree to which samples will be multiplexed must be planned into the design to balance read depth (and thus higher confidence in calls) versus the cost of the assay, since higher read depth leads to lower multiplexing capacity and thus increased per sample assay cost (43, 57, 58). Ensuring that the assay design and bioinformatics analysis take into account the region’s characteristics, it should be applicable to individual assay developers building Dx assays on other platforms as well. Finally, it is important to develop models that take into account the expected sample throughput, frequency of testing, the assay TAT, and the degree of batching to forecast the optimal multiplexing strategy. For batching samples there must exist guidelines for standard multiplexing and read depth to ensure equivalence of test results.

### Quality control standardization

The lack of industry-wide standardization of critical components of QC also represents a challenge for CDx development. The current NGS technologies have higher error rates and novel error modes compared to traditional sequencing, which results in variability in mutation reporting (59–61). Thus, during test development it is essential to have a strategy to detect and reduce the frequency of false positives and then to establish QC procedures to assess test performance, yet there is no established or generally accepted approach (62, 63). This strategy will likely involve varying bioinformatics parameters of the variant calling software and establishing a method to confirm mutation calls with orthogonal methods. Investigating false positive calls is crucial during assay development and refinement. While Sanger sequencing is still considered the gold standard, its lower sensitivity of detection [around 17–25%; (64)] limits its use for confirming mutations at the low frequencies that are commonly detected with NGS. Multiple strategies for orthogonal validation are possible, such as using a different assay design on the same NGS platform to evaluate design robustness or employing an orthogonal NGS platform with similar sensitivity to identify any platform-specific artifacts. Orthogonal validation with non-NGS platforms such as Sequenom, COLD-PCR, and pyrosequencing may be a preferable approach and these are also gaining popularity as clinical NGS validation strategies (44, 46, 65). False negative calls are more difficult to detect but the utilization of variant call files (VCFs) that report read depth at every position allows for positive confirmation of a wildtype call and not just the absence of a variant call at that position. Second, standardized procedures for QC, including spike-in sequences are yet to be standardized. Some have proposed that spike-in samples should mimic the region of interest in terms of genomic region tertiary structure, interfering genomic regions competing for similar priming sites and, lastly, for genomic complexity, including but not limited to base distribution, presence of similarly presented homopolymeric regions or the known regions of ambiguity such as GC combinations that have been found to complicate variant analysis in a platform-specific manner (29). Recent forums for NGS standardization (43, 44) have discussed the needs for both artificial sequences, which will allow quality assessment of library preparation and analysis (66), and clinically relevant biological mimics, which can faithfully recapitulate biological variation induced by genome complexity as well as serve as a good benchmark for matrix-associated artifacts, e.g., FFPE matrix artifacts. Without industry-wide recommendations or guidance from regulatory authorities, this aspect of CDx development represents a challenge.

### Clinical and diagnostic RNASeq assay design challenges

The use of RNASeq for transcriptional profiling, gene expression studies, identification of variants, and pathological fusion or splicing events (67) is an area of great interest to the clinical genomics community. Clinical RNASeq brings to the fore the capacity to utilize gene expression signatures for highly informative disease sub-type classification or prognosis signature development, as has been demonstrated by gene expression based Dx tests like Agendia’s MammaPrint test (68) or Genomic Health’s OncotypeDX tests (69). Clinical RNASeq at the whole transcriptome level offers invaluable insight into a patient’s transcriptome and associated gene expression changes informative of pre-disposition to cancer or patient stratification strategies. It is especially pertinent for conditions where alternative splicing and isoform selection can affect response to drugs or can predict selective outcomes in response to therapy. RNASeq analysis can be used to develop a robust molecular sub-type signature for a cancer as is apparent from recent studies utilizing gene expression signatures for prognostic and Dx assays (70, 71). In reality, as with issues facing the whole genome sequencing and whole exome sequencing field, it is more likely that targeted panels rather than whole transcriptome offerings will first show clinical utility.

Some of the issues that hinder the adoption of clinical RNASeq are the quality of the RNA from clinical biopsy materials, extremely complex bioinformatics and statistical analysis as well as design of the experiment and its execution in the clinic. The quality and quantification of RNA is critical for successful library preparation and QC controlled analysis of the sample. Clinical FFPE sample-derived RNA is likely to require pre-processing repairs or methodologies to enable low input amplification or enrichment based library preparation. Sample RNA preparation and RNASeq process reproducibility and accurate quantification will have to be highly validated to avoid issues such as prep based biases in quantification of GC-rich transcripts or small RNA species. It will also be important to assess the impact of factors such as RNA secondary structure, the presence of small RNAs in the sample or interfering substances (72). Any lack of read-out reproducibility in a gene-specific manner will hinder the establishment of fold change cut-offs for clinical decision-making (73). Qualifying adequate depth of coverage is critical because accurate quantification of transcripts in clinical RNAseq is dependent on read depth (74).

The bioinformatics analysis of RNASeq in the clinic is considerably more complex than pipelines for DNASeq. For one thing, normalization of data needs to be highly accurate for the technology to be quantitative for the measurement of relative expression values (75). As algorithms for non-clinical RNASeq are improved and as scientists employ better controlled experiments and statistical strategies (76), some of the issues that have plagued clinical RNASeq bioinformatics may be resolved in the near future. Definition and standardization of clinical databases and annotation pipelines is another critical requirement for clinical RNASeq. Currently, because of variability in gene models in different databases such as AceView and RefSeq as well as frequent changes to the databases, non-clinical RNASeq efforts encounter high variability in definition and annotation of regions. In addition, one of the key features of clinical RNASeq will be the ability to identify specific re-arrangements and spliced isoforms. Considering that detection of fusions and gene re-arrangements have high clinical relevance, it will be necessary to develop both bioinformatics methods and mate pair library construction protocols or similar technology but simpler workflows to detect re-arrangements and gene fusions (77). The design of targeted experiments should enable more hypothesis-free quantification of the staggering complexity of gene fusions and transcript re-arrangements possible as well (78). Without such a highly complex identification and quantification strategy the power of clinical RNASeq cannot be fully realized. Targeted RNASeq approaches, particularly with amplicon-based panels, would need to have highly plexed designs to allow a more discovery oriented capture approach while allowing highly sensitive quantification. Hybrid capture based panels would possibly offer more robust splice isoform coverage but suffer from more labor intensive protocols.

Reference materials, controls, and QC standards need to be defined for clinical grade RNASeq in the same way these are becoming standardized for clinical DNASeq. An advantage for the clinical RNASeq field is the availability of the highly qualified human reference MAQC-A and MAQC-B reference materials and the extensive data on tissue-specific expression of potential housekeeping genes from exhaustive microarray profiling (79). This approach has been utilized to test and aid data correction in RNASeq in research settings and may find easy integration into clinical practice as well (80). Recently, the set of eukaryotic mRNA mimic Spike-In Control Mixes developed by the External RNA Control Consortium (ERCC) has been suggested as a clinically useful control option. These have pre-formulated quantified blends of 92 transcripts derived from National Institute of Standards and Technology (NIST)-certified DNA plasmids. The call for a MAQC-like platform comparison for RNASeq to identify issues and to evaluate platform-specific biases or strengths is being addressed by at least two consortia, the FDA’s SEQC (MAQC-III) group and the Association of Biomolecular Resource Facilities – Next-Generation Sequencing (ABRF–NGS) group study. These results will be highly informative to the developers of clinical RNA sequencing (RNA-Seq) assays.

An emerging theme in the translational NGS community has been the utilization of RNASeq for detection of mutations (81, 82). Analysis pipelines that can account for factors like editing biases are not publicly available or are not sufficiently validated to allow such analysis in a clinical context, but once achieved these may offer a highly efficient method for capturing both mutational and expression level information in the same analysis (24, 83). Increasingly, studies that compare the benefits of both types of studies in combination with even epigenetic and microRNA signatures of the tumor for comprehensive profiling are likely to gain traction. The use of RNAseq instead of clinical DNASeq is likely to require a significant effort that includes matched RNAseq–DNAseq analysis and the development of sophisticated algorithms for analysis. Nonetheless, it appears likely that for at least certain molecular sub-types RNASeq-based gene expression profiling and analysis may provide a more predictive result than mutation based analysis alone.

## Post-Analytical Challenges

### Bioinformatic mutation calling algorithms

**Figure 1:**
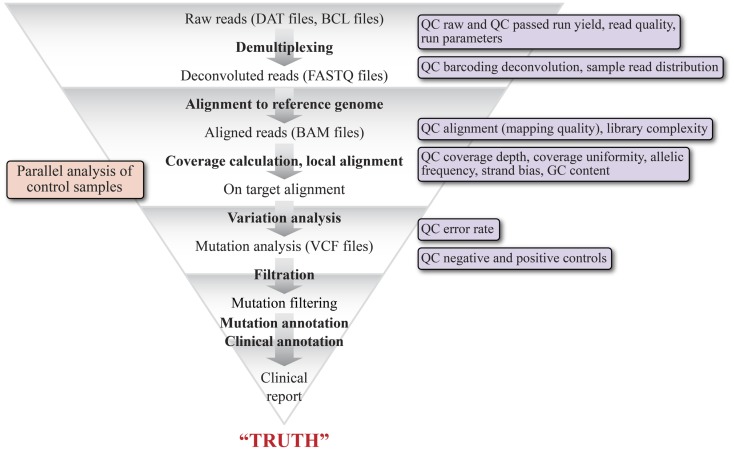
**Schematic representation of the various bioinformatics and statistical analysis steps of a typical clinical NGS variant detection data pipeline**. The graphic illustrates the major modules of the pipeline and their output file types, beginning with raw reads (DAT files) and ending with a clinical report. The pipeline is highly tunable, as each of the steps can be optimized by adjusting parameters specific to each step. The triangular shape is intended to convey that each step acts as a filter to remove reads that do not represent variants. The key quality filters that can be applied are shown in the boxes to the right.

One of the major hurdles to adoption of NGS for CDxs is the current state of variability in the performance of variant calling software depending upon the bioinformatics pipeline used (84, 85). It is a routine occurrence that variations in mutation detection are observed from the same raw data set when utilizing different algorithms for variant calling, even with the assumption that similar pre-variant calling processing was performed on the final dataset (86). Figure 1 is a high level schematic illustrating the basic steps in a bioinformatics pipeline to stress the number of steps and the complexity of variables that impact mutation detection. The initial sequencing data (DAT files) are derived from Illumina imaging data or Ion Torrent pH change related voltage data. Basecall (BCL) files contain data where the sequencing data (images or voltage) have been translated into a nucleotide call. Multiplexed data are then separated into per sample data via the sequencing index identity and FASTQ files are generated, which contain sequencing read data that include the sequence and an associated per base quality score, called a phred score or Q score (87, 88). Reads are then aligned to a known reference sequence containing genomic coordinates and organized into BAM files (89). Variation analysis, or variant calling, refers to the assignment of non-reference status (i.e., a mutation or a variant) to a specific queried position in the genome and generates a tab separated VCF. The variant calls are filtered to minimize false positives and false negatives while maintaining the sensitivity and specificity of the data by utilizing the phred quality scores, which vary on different platforms (63). To generate a clinically actionable report, the high confidence variants are unambiguously annotated based on clinical data showing a causal relationship between the variant and disease and with information about the variant in the literature (90, 91). A vast variety of software is available for each step of NGS data analysis, as are a number of bioinformatics suites designed specifically for Dx testing and which can be tailored to provide a streamlined, module locked analysis for Dx processing (63, 91). Some suites may also allow the user to change settings for test development purposes. Recently, the NIST spearheaded an effort (92) to develop a highly confident variant caller by encouraging the NGS community to share sequencing data of their NGS reference material NA12878 (v.2.15). This effort should greatly aid the standardization of analysis methodology and better QC for assessing false positives and false negatives (66, 92).

The traditional regulatory framework makes integration of the NGS data analysis software into the Dx device system imperative, with a fixed version of the analysis algorithm for the regulatory submission. This presents a challenge for the device developers since variant calling software applications are continually evolving, particularly in the ability to detect indels, in efforts to reduce analysis time and in the use of control set parallel analysis (41, 85, 86, 93). As new versions of variant calling software with better sensitivity and specificity become available, it is reasonable to assume, based on current precedent, that new 510(k) submissions will be required for these devices.

Standardization of data QC and filtering, variant detection and annotation of samples is imperative for developing Dx tests. Ideally, NGS-based data analysis should be subjected to rigorous internal and external QC with rules to accept or reject data akin to Westgard rules (94, 95) used for other analytical tests. The field is still open for discussions on how these rules should be implemented for NGS-based CTAs and Dx tests. For example, are traditional Westgard rules applicable to a quantitative parameter of NGS-based mutation detection tests such as mutation allele frequency? If not, then what type of quantitative rules can be used to establish in control processes? It is imperative for the field to define the type of control samples and the QC procedures to accept or reject runs. Some laboratories argue that internal control targets must also be met prior to a decision to report mutations (43, 85).

Another novel aspect of NGS mutation calling is that variants are rated based on the certainty of the call (87, 88). Phred quality values are assigned to specific steps in the process such as base calling and read alignment. Read depth, read quality, frequency of detection of the allele, strand bias, annotation as germline variant or variant of unknown significance, or lack of “actionability” all can be used to assign a confidence score to a particular call (57, 89, 96). Segregation of variants per characteristics of read depth, base quality, read quality, and strand bias are easily automatable with most Dx instruments available, but current software programs do not provide easy readout of mis-alignment-based read drops, reads that are exempted from final analysis by homopolymer-based inaccuracies, reference allele bias, or reference genome bias (60, 61, 97). These are post-analysis computing requirements that still need to be built into software to minimize operator involvement.

It is interesting to note that each sequencing platform has its particular advantages and drawbacks in terms of regional biases that complicate variant calling. In the past, Illumina MiSeq data have been associated with high accuracy but increased strand bias with GC-rich motifs, as well as low accuracy for homopolymer stretches beyond 20 bp (97, 98). In the November 2013 FDA 510(k) Decision Summary for the MiSeqDx instrument (Number k123989), Illumina specifically claims the ability to detect single nucleotide variants (SNVs) as well as deletions up to three bases. Based on a very limited data set, the instrument can also detect 1 bp insertions, but this is limited to non-homopolymer regions, since the MiSeqDx instrument was shown to have problems detecting 1 bp indels in homopolymer tracts, e.g., polyAs. The notification also states that Illumina’s current MiSeqDx analysis software will automatically remove any homopolymer tracts of longer than eight continuous identical bases (R8 error). Interestingly, the MiSeqDx instrument claims to be a qualitative detection platform rather than quantitative. The MiSeq has generally been reported to have higher fidelity for indel calling than Ion Torrent (28, 61, 99). Ion Torrent homopolymer regions beyond 20 bp tend to be misaligned and discarded so that alignment algorithms must be optimized per region of interest to allow inclusion of misaligned regions (32, 61). The Ion Torrent Dx platform specifications will become clear when it is registered. Strand bias related inaccuracies and decreased depth of coverage or uneven coverage (due to allele dropout in case of sampling error or as a function of tumor heterogeneity) can also compound the problem of mutation calling inaccuracies. Accurate base calling algorithms for Dx assays must minimally utilize spike-in controls during technical feasibility experiments and raw data controls for software training that include mutation calls in regions of predicted poor base calling if those are part of the assay design (41, 43, 66). The use of a highly sequenced reference sample, such as NA12878 by NIST (v.2.15) for software training and algorithm development has been proposed in many forums such as the NIST “Genome in a Bottle” Consortium (92). Recently, the same was used by Illumina to demonstrate accuracy in its MiSeqDx platform 510(k) submission application. Additionally, it is reasonable to propose to include engineered mutations as part of spike-ins where inaccurate calls may result due to biases from GC-rich motifs, strand bias, reference allele bias, homopolymers, and regions of low coverage if down-sampling total calls for normalization, etc. For assessing the accuracy of the data pipeline, normal/reference sample pairs may be developed as proficiency testing (PT) material. Alternatively, specially designed artificial DNA mixtures that contain the majority of expected mutations (from literature and clinical findings) should be used as reference material in accuracy, sensitivity, and precision studies in the technical feasibility phase. The National Cancer Institute (NCI) initiative to make specific mutations available as plasmid constructs as well as the availability of characterized mutant DNA or recombinant tissues from companies like Horizon Dx are allowing test developers to devise such experiments with spike-in-based QC (43, 66). From its recent guidance on Personalized Medicine, the FDA also seems to acknowledge that testing of variant calling for a specific set of mutations and the establishment of the platform’s sensitivity and specificity may be sufficient for the clearance of a NGS-based regulated device. One novel aspect to the application of NGS-based tests is the need for a standardized set of raw data for mutation calling algorithm development. To meet this PT need, the NIST Genome in a Bottle Consortium as well as CAP have both been actively advocating availability of public data sets from extremely well studied samples as PT material to assess a particular pipeline’s sensitivity and specificity in mutation detection to avoid lab to lab variation in mutation detection.

**Figure 2:**
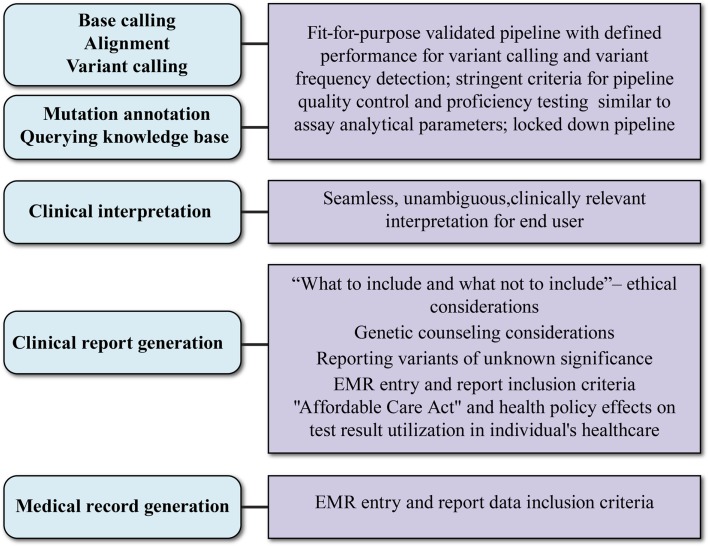
**Aspects and key considerations of clinical NGS data reporting**. Main aspects of clinical data reporting are shown in ovals to the left; key considerations are shown in boxes to the right. The uppermost three aspects rely on the bioinformatic pipeline. What test results are reported in the clinical report (fourth oval) is influenced by socio-ethical considerations and may require genetic counseling and support systems. The evolving payer landscape and medical records guidance will affect how NGS clinical reports are captured in patient records.

In addition to bioinformatics analysis for variant calling, there are several aspects of data interpretation and annotation that must be standardized for NGS tests to be adopted into clinical practice. These are graphically represented in Figure 2 and are discussed below.

### Data reporting

If the FDA requirement for a NGS-based Dx approval is demonstration of accuracy and precision for each assayed base, it is possible that Dx developers may choose to limit the reportable content of a NGS panel by utilizing base masking in an effort to reduce the extent of analytical validation efforts. In the recent 510(k) application for the MiSeqDx instrument and the CFTR gene Dx test on the instrument, data showing the orthogonal validation of a subset of base positions was accepted, suggesting that the FDA may only require a sponsor to show performance data for the unmasked, reportable nucleotide positions on future submissions of panels or single-gene assays. It will be interesting to note the Agency’s guidance on this topic since the masked data could potentially still be utilized for analysis to develop or enhance predictive mutation signatures on retrospective analysis.

Another key consideration for data reporting is the reporting of variants of unknown significance. The ACMG guidelines from 2008 (100) defined various cases of variants of unknown significance including: (1) previously unreported variations with possible ramifications for the disease being studied. This includes indels, frameshift mutations, and invariant splice site AG/GT nucleotides variants that can alter the reading frame and thus the expressed gene product. (2) Previously unreported variations that may or may not be causative of the condition. These are exemplified by missense changes, in-frame indels, and splice consensus sequence variants or cryptic splice sites that may affect regulatory processes, e.g., interruption of splicing enhancers or suppressor sites. In these cases, clarification of the clinical significance of variants is required and it may be important to flag them accordingly in a report. (3) Previously unreported variations that are probably not causative of disease, e.g., synonymous mutations that do not alter protein sequence or affect processing or regulatory pathways, or are found in addition to a variant known to be associated with pathologic change (in autosomal dominant disorders). (4) Previously reported sequence variations that are recognized as neutral variants with evidence available that the variation has been consistently observed in a normal population and does not associate with disease or predisposition to disease. (5) Sequence variation not known or expected to be causative of disease, but is found associated with a clinical presentation, e.g., variants that contribute to disease as low-penetrance mutations which alone or in combination may or may not predispose an individual to disease or modify the severity of a clinical presentation in complex disorders. For such a category the institute suggests reporting as not definitive mutations and stating that medical management decisions should not be made on the presence of the variants alone. This last is probably the most efficacious solution for reporting NGS-based variants of unknown significance since it allows capturing of the profile without unduly triggering medical actionability. Unfortunately, the current forms of patient consent are usually quite limiting and restrict public sharing and analysis of data utilizing big data analytics. There is clearly a need for patient consent agreements to allow meta-analysis, but this is the topic of the next section, data privacy in the age of big data analytics.

Reporting of incidental or serendipitous findings is another area of complexity for NGS-based tests. Some are proponents of the idea that incidental findings should not be reported at all in clinical sequencing without strong evidence of benefit, while others advocate that any and all variations in disease-associated genes are potentially medically useful and therefore should be reported (2, 17, 41, 44, 46). Recognizing the difficulties of reporting such secondary findings which are medically important but unrelated to the reason for test ordering, the ACMG constituted a special Working Group on Incidental Findings in Clinical Exome and Genome Sequencing to make recommendations for addressing such findings in pretest patient discussions, clinical testing, and the reporting of results (101). In the case of targeted oncology panels, this may not be an issue unless specific loci are associated with enhanced risk for other conditions or where particular polymorphisms can affect existing health care routines and drug regimens. Currently, the ACMG working group has only recommended reporting those incidental findings for which preventive measures or treatments are already available or for disorders in which patients are asymptomatic despite the presence of pathogenic mutations. Generally, the recommendation was to report pathogenic variants as incidental findings, e.g., those where the “sequence variation is previously reported and is a recognized cause of the disorder” or “sequence variation is previously unreported and is of the type, which is expected to cause the disorder” (100). These two were chosen no doubt because the group recognized that attempting to report and interpret variants of unknown significance as incidental findings would be particularly challenging. The report also stressed that identification of monogenic diseases via a clinical NGS panel as an incidental finding is highly improbable by current practice.

### Privacy of and access to patient results

Ever since the report that individuals could be identified from anonymous NGS data (102), privacy groups have been justified in their concerns about having sensitive data made public as a result of inappropriately controlled data and reports. Privacy of patient results is also linked to maintaining the highest standards for patient consent to NGS-based testing, anonymized data generation, secure data storage, encryption, and transfer processes that meet the highest standards data (103). The converse of this concern relates to the data that reported back to the patient, especially incidental findings unrelated to reason for which the test was performed. In contrast to whole genome sequencing, oncology-based panels are focused on tumor specific genes assessed in the context of the tumor. They have less content with associated incidental findings and thus are less likely to trigger traditional socio-ethical impact (104). However, an issue which lacks resolution is the reporting of low frequency mutations for which the allele frequency based drug action has not been studied. For example, the technical sensitivity of an assay may allow the detection of a mutant at 0.1%, but there is no framework with which to interpret such a finding, and reporting it to the patient may cause more harm than good.

### Interpretation of results

The mainstream adoption of NGS Dxs will rely heavily on easily interpretable test results. One critical aspect of data interpretation with NGS-based tests is the comparative reference human genome. This is an individual genome and may not be an ideal reference genome for most individuals in the population. For this reason, some commercial NGS providers have started stressing the need for a matched germline control comparator sample such as peripheral blood or normal adjacent tumor tissue from tested individuals. The constant evolution and enhanced annotation of the reference genome as sequencing-based studies continue to reveal new genomic complexities also confounds interpretation. In the example from the MiSeqDx 510(k) decision summary, it is interesting to note that a compound reference genome derived from two well-characterized samples was utilized in addition to human genome build 19 [NCBI Human reference February, 2009 (GRCh37/hg19) assembly] [FDA 510(k) K123989 decision summary]. For example, the two genomes differed in a particular homopolymer run, which was a run of 14 A’s according human genome 19, while the sequence in the composite reference genome had a run of 15 A’s. This was significant because it directly impacted interpretation of the MiSeqDx sequencing accuracy study, since all 13 samples analyzed were reported as having 1 bp insertions since 15 A’s were detected in all 13 samples. As new variants and polymorphisms are identified, it may be warranted to re-annotated or re-issued reports to include the new data or its new interpretation.

## Overview of Diagnostic Test Regulatory Approval Process

**Figure 3:**
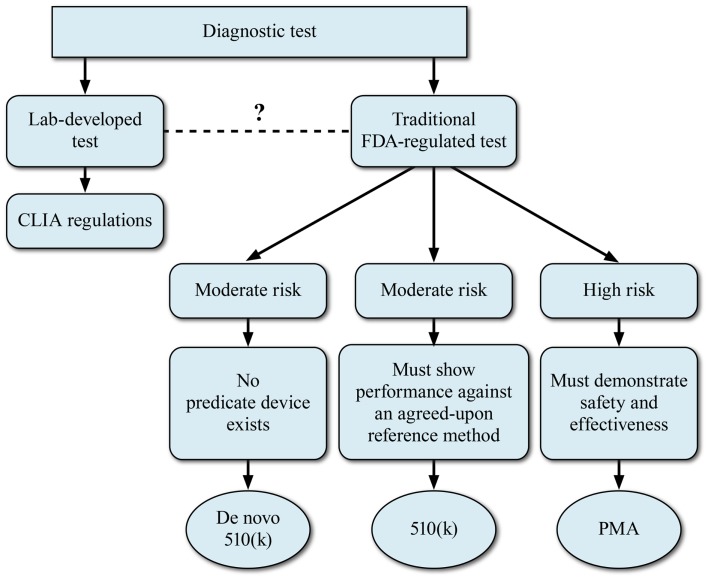
**Regulatory models for development of NGS-based diagnostics**. The FDA device classification for a regulated NGS-based diagnostic device will depend on the perceived risk associated with the diagnostic device.

As a prelude to the regulatory challenges, we digress to provide an overview of the Dx test regulatory approval process. The basic regulatory pathway options for Dx device development are summarized in Figure 3. This section describes a generic IVD submission process with the authors’ comments on possible paths for NGS-based devices.

For any given test that is submitted for FDA consideration, the route to commercialization may be via a 510(k)/pre-market notification process or via a PMA application. The decision to take a NGS-based clinical test via the 510(k) or PMA process will depend largely upon the perceived risk associated with the Dx device. The 510(k) Dx IVD process relies on the presence of a predicate device or devices. However, FDA has utilized the *de novo* 510(k) pathway when the risks of the new device are consistent with other 510(k)-cleared devices but a clear predicate is not available. The 510(k) process may be appropriate for those NGS-based tests that will be utilized for monitoring disease or for tests where the perceived risks are lower. Although the concept of a predicate device is woven into FDA’s device regulation, the reality for the genetic tests that have been cleared or approved to date is the new system is not compared head-to-head with a previously cleared system. Rather, the new method is compared to a gold standard method, which is considered truth. For most DNA applications, the gold standard has been bi-directional Sanger sequencing. Applications which have relatively higher perceived risk to the patient, such as NGS-based oncology tests, will likely be required to go the PMA route to demonstrate safety and efficacy. In these cases, a reference method will also be used to demonstrate accuracy of the device.

A PMA submission for a CDx NGS test will entail coordinated review of the drug by the Center for Drug Evaluation and Research (CDER) and of the device by the CDRH (or CBER for certain disease indications). The IVD developer will have to demonstrate the safety and effectiveness of the *in vitro* Dx device when used as specified in the label. The Dx device must be considered as an entire Dx system including reagents, hardware, software, data analysis, and result reporting. Use of the device in the pharmaceutical clinical trial will provide important data to demonstrate clinical validation of the assay. Although NGS IVD submitters may have to undergo an advisory panel review regarding clinical, regulatory, scientific and statistical issues due to the novelty of the NGS platform and assay structure and readout, it seems doubtful since other CDx applications have not had this hurdle and FDA has seen fit to clear the Illumina platform with no such advisory panel requirement. For an approved PMA any modifications to the test or device, manufacturing process, its labeling, intended use or sensitivity or specificity would require FDA notification and prior approval. In general, it is imperative that NGS-based Dx stakeholders seek clarity utilizing pre-submission meetings with the CDRH, and specifically the Office of *In vitro* Diagnostics and Radiological Health (OIR), well in advance of trial planning. It is important to engage in such discussions early as FDA thinking is evolving rapidly.

Many of the regulatory challenges for CDxs are not unique to NGS. Although NGS tests may be more complex than other technologies, the same principles will apply. The FDA’s expectations on the analytical validation and performance characteristics of NGS-based assays will differ somewhat for each individual assay. However, the 510(k) clearance of Illumina MiSeqDx reveals some aspects of the regulatory agency’s viewpoint on validation. Since this is the crux of the regulatory challenge, we summarize in detail the main aspects of Illumina’s 510(k) submission studies [510(k) summary, e.g., K124006, November 2013] as early pointers to the type of experiments FDA may expect.

### 510(k) clearance of Illumina MiSeqDx

With the MiSeqDx clearance, the FDA has given some indication the type of information that will be required for approval a NGS-based CDx for tumor mutation status. First, the 510(k) summary indicates that accuracy data for all claimed specimen types and nucleic acid types were required. Two sources of well-characterized samples (based on well validated sequencing methods) were queried with all of the claimed sequence variation types, types of sequencing and with the sequences located in varying sequence context (e.g., different chromosomes, GC-rich regions). The 510(k) summary indicates that sequence data generated with a sequencing technology platform and variant calling method independent of the device manufacturer is required for at least one of the reference samples. Percent agreement and percent disagreement with the reference sequences were described for all the regions that were queried by the instrument. Illumina performed accuracy testing in three studies. The first assessed overall accuracy over a wide portion of the genome by utilizing 13 very well-characterized samples from parent–child triads that had been sequenced by multiple laboratories and multiple sequencing technologies. Human reference genome 19 was used to assess accuracy across 24,434 bases on 19 chromosomes encompassing a variety of genes containing potentially clinically relevant exons. The second study assessed the accuracy of the MiSeqDx instrument at 17 highly confident variant calls in the NIST NA12878 standard reference material. The third accuracy study assessed the instrument’s performance in detecting small insertions and deletions by analyzing six samples using the Cystic Fibrosis 139 Variant Assay, which included a subset of clinically significant indels in *CFTR*. The detected insertions and deletions were all confirmed with bidirectional Sanger sequencing as the reference method. Such accuracy studies helped Illumina define its performance specifications for homopolymer stretches, nucleotide repeat regions, and ability to detect indels.

For precision/reproducibility studies, the 510(k) summary indicates that data should be generated using on multiple instruments, with multiple operators and at multiple sites, and that performance data are required for all claimed specimen types, nucleic acid types, sequence variation types, and types of sequencing. As discussed in the Assay Design, a special emphasis was given to variants located in varying sequence context, such as different chromosomes and GC-rich regions, along with a requirement to utilize a high confidence reference sequence data. To this end, Illumina performed three precision studies. For the first study, 13 well-characterized sequenced samples were analyzed in 9 runs using 3 different MiSeqDx instruments and 3 different operators. Samples NA12877 and NA12878 were run in duplicate to assess repeatability. Ninety-four samples and two non-template controls were tested across three lots to establish lot-to-lot reproducibility of the Illumina universal reagents. Each lot was split into two 48-sample runs to test reagents and all possible index primer combinations. All sequencing runs were completed by a single operator and on a single MiSeqDx instrument to remove potential variance contribution from operator or instrument. The MiSeqDx Cystic Fibrosis 139 Variant Assay reproducibility study involved a blinded study with three trial sites and two operators per site. Two well-characterized panels of 46 samples each were used for testing. These contained a mix of genomic DNA (gDNA) from cell lines with known variants in the *CFTR* gene and variant containing cell lines spiked into leukocyte-depleted blood to assess variability from the gDNA extraction steps.

Illumina also addressed the issues of sample cross-contamination (carryover) and intra-run performance. For intra-run performance, a 48-sample library of two samples with unique variants arrayed in a checkerboard of an alternating high concentration (500 ng) and low concentration (100 ng) input was utilized. For inter-run carryover 2 libraries were prepared each with 47 replicates of a single gDNA sample and 1 no template control (NTC). The samples were unique in each library and continuous run assessment was performed to demonstrate absence of carryover. The reproducibility and accuracy of multiplexing was also tested with 12 indices (barcodes) per sample sequenced. Accuracy for all sample/index primer combinations was confirmed as 100% by Sanger bi-directional sequencing and PCR-based confirmation.

For testing the contribution of common interfering substances to variability, four endogenous interfering substances (bilirubin, hemoglobin, cholesterol, and triglycerides) were spiked in eight unique whole blood samples. Blood collection variability and gDNA sample preparation variability were also evaluated, along with sample input amounts, thermal cycler effects, and sample stability. DNA extraction methods were assessed using 168 specimens (14 samples × 2 operators/extraction method × 3 runs/operator × 2 replicates/extracted gDNA sample).

The MiSeqDx approval gives insight into some of the regulatory expectations for NGS-based assays and is summarized here with some general headers for reader clarity:

Specimen and processing-related validation:

(i) The specimen type(s) as source of nucleic acid.
(ii) The type(s) of nucleic acids (e.g., germline DNA, tumor DNA).
(iii) The nucleic acid extraction method(s).

Sequencing variation-related validation:

(i) Type(s) of sequence variations (e.g., SNVs, insertions, and deletions).
(ii) Type(s) of sequencing (e.g., targeted sequencing).
(iii) The read depth required for the sensitivity being claimed and the validation data that supports those claims.
(iv) Accuracy and precision of the test and the types of sequence variations that the test cannot detect with the claimed accuracy and precision (e.g., insertions or deletions larger than a certain size, translocations)
(v) The upper and lower limit of input nucleic acid to achieve the claimed accuracy and reproducibility.

The MiSeqDx instrument’s current *de novo* classification is for qualitative assessment for profiling of peripheral whole blood samples, which tend to be of a higher quality. It is important to note new tests, including CDx devices, on the platform are likely to require PMA submissions, especially for tests utilizing heterogeneous samples like tumors. The current MiSeqDx clearance for qualitative results opens the discussion on what further validation strategies may be required to achieve quantitative detection of mutations (e.g., quantitative allele frequency), which may be one of the strengths of clinical NGS.

## Unanswered Answered Regulatory Questions

### How many mutations will have to be clinically validated?

The FDA has hinted at possible accuracy requirements for complex, multi-analyte specific assays, genes, and panels at the DIA Meeting on Personalized Medicine and CDxs (November 6, 2013). This provides important insight for CDx applications involving tumor suppressor genes and certain oncogenes since actionable mutations may occur anywhere along the length of the gene. FDA has suggested three potential strategies:

- Sequence clinical samples from the intended use population and compare to reference method results.
- Sequence procured samples that span the relevant classes of variants and compare to reference method results.
- Sequence well-characterized reference sample(s) and compare to reference sequence.

### Can an NGS multi-gene or multi-transcript panel be approved as a diagnostic platform, allowing multiple CDx submissions?

At the 2012 Friends of Cancer Meeting the FDA publicly indicated their interest in reviewing NGS-based cancer panels similar to the panels that have been cleared as microbiology devices (i.e., devices that detect multiple viruses and bacteria in a single product) (105) (focr.org). Although the details of this type of submission would need to be worked out between FDA and an individual sponsor, it seems likely that some level of clinical evidence would be needed for each gene or mutation included on the panel. It is possible, similar to the cystic fibrosis assays, that this list could be developed based on medical input and literature. From that point, more specific claims about individual genes could be made on a gene-by-gene basis including CDx claims if the product has been used as part of a clinical trial investigating a particular drug. It is likely that any cancer panel would be subject to a PMA [rather than a 510(k)], and amendments to the original PMA with additional claims on a per panel member basis would be a rational approach to updating the claims for each new CDx.

### How will existing genomic diagnostics align with approved NGS-based diagnostics?

Currently, the intended use statement for each of the Dxs that have been approved in conjunction with a drug list the drug name in the intended use statement. It is reasonable to expect that this policy will continue and that in order for a drug and Dx to be co-marketed the drug and device will need to be linked. Even if there are multiple devices available for testing in conjunction with a specific drug, any of the approved devices will be allowed.

### What is the appropriate orthogonal technology?

What is the true measure of truth when comparing discordant results? FDA has shown with the recent Illumina clearance that they expect NGS-based mutation calls to be confirmed by an orthogonal technology (in many cases bi-directional sequencing). However, disagreement exists within the NGS community as to what is true orthogonal validation of a NGS-based mutation call (17, 64, 106). The enhanced sensitivity of mutation detection down to 1–5% allele frequency implies that orthogonal validation will require a platform with similar sensitivity. While Sanger sequencing is being used to support mutation validation, for example in the Illumina MiSeqDx 510(k) clearance, it is not possible to use Sanger data to provide a definitive call when mutations in the range of 1–15%. Generally, if NGS and Sanger give discordant results labs tend to use tie breaker tests such as pyrosequencing or Sequenom-based sequencing on the MassArray system. Both of these technologies can detect mutant allele frequencies down to 5–10% frequency and are finding increasing usage in NGS validation. As Sequenom and pyrosequencing vendors create niche products tailored for NGS validation these will likely integrate into clinical NGS workflows. The FDA has shown flexibility in allowing use of these types of technology as orthogonal methods when Sanger is not sensitive enough. However, the FDA will insist on appropriate validation of these methods and will expect to review these validation packages as part of the review process.

Another approach, likely to be costlier but with the opportunity to have near equivalent sensitivity of detection, is the utilization of a second NGS technology for confirmation of assay results, e.g., utilizing both the Illumina and Ion Torrent platforms where the difference in underlying technology make a confirmation of positive results quite robust. The main issue to be cognizant of is the need to adjust analysis parameters to provide equivalent performance with respect to mutation call sensitivity, since each platform uses its unique quality score for data quality assessment. For example, while Ion Torrent recommends using a phred value of Q20 (99% specificity) for high confidence variant analysis, Illumina recommends at phred value of Q30 (99.9% specificity) for ensuring high confidence calls (61). The difference in acceptable phred scale values arises from differences in platform technology, related background signal and noise calculation algorithms (107).

## Strategic Challenges for Drug and Diagnostic Developers

**Figure 4:**
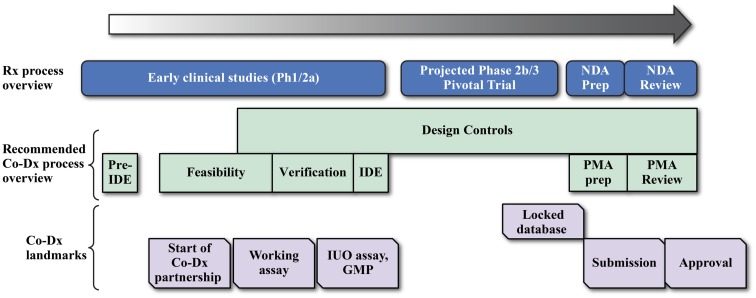
**Coordination of drug and device development for a successful companion diagnostic submission**. Drug companies and diagnostic developers may work together in several different cost sharing and assay development landmark payment formats for the development of the final IVD product.

Developing any CDx can be enormously challenging, as seen in the development of the *BRAF* mutation (108) and *ALK* gene fusion (109, 110) tests. A primary reason is that the device development timeline does not align with the drug development timeline, as illustrated in a development timeline chart (Figure 4) (15, 108, 111). Ideally, CDx development for the NGS assay would start with the initiation of early phase studies (Ph1/2a studies in Figure 4) to allow sufficient time for development of the Investigational Use Only (IUO) version of the device before start of the phase 3 pivotal trials. But this is not often the case, and compromises and work-around strategies are sometimes necessary. Thus, in another example of navigating the rapids, pharmaceutical and Dx companies face some unique challenges in NGS-based CDx development, which are summarized in the next sections.

### Drug development challenge 1 – is a companion diagnostic needed?

The first challenge is whether a co-Dx test is in fact required and how a multiplexed RNA- or DNA-based NGS panel would fit into the traditional CDxs scheme. While a CDx may uniquely position the drug in the marketplace, the overarching reason for developing a CDx is because it is required for the drug approval. The current FDA guidance dictates that if the test is necessary for the safe and effective use of the drug, then a co-Dx is required. The key factor to determining whether a CDx is required is the efficacy of the drug in a biomarker negative population. If efficacy in the biomarker negative population is sufficient for drug approval, then a CDx may not be required, at least for an initial approval. Thus, this question should be answered early in drug development (12, 109). It appears that NGS-based CDxs will be more relevant in the near future in certain oncology indications where genetically targeted therapies are currently prevalent, such as lung cancer, breast cancer, and colorectal cancer.

### Drug development challenge 2 – use a single-gene assay or multi-gene panel?

At one level, the question seems to challenge one of the guiding principles of Dx development: the simpler the better. Analytical validation of a multi-gene assay, as discussion elsewhere in this article, will undeniably be more work than validation of a single gene. Yet, it might be necessary to consider the pursuit of the multi-transcript or multi-gene panel in some cases such as if the predictive biomarker is a set of mutations in the genes on the panel, i.e., if the marker is a signature for response rather than a single Dx gene mutation. The multi-gene panel approach is predicated on two assumptions: (1) that the FDA will permit the sponsor to mask data from genes that are not required for safe and effective use of the companion therapeutic, and (2) that the FDA will permit different levels of rigor in the validation of genes on the panel, based on whether they are necessary for safe and effective use of the companion therapeutic. The authors firmly believe that the multi-gene panel is a step toward the “multi-gene Dx/many drugs” model even though the path there is not obvious. One of the reasons that the change from “one-drug/one-gene Dx” model to the “multi-gene Dx/many drugs” model will be so disruptive is that the test results from a multiplexed panel could actually lead to the use of a competitor’s drug. This leads to the next challenge of how best to design clinical studies to best take advantage of all the content on the NGS assay.

### Drug development challenge 3 – optimal trial design for NGS-based diagnostics?

By definition precision medicine focuses on a subpopulation of patients expected to respond to a given therapeutic. Sometimes the population can be quite small, as in the case of metastatic lung cancer patients with the ALK gene fusion, for which crizotinib is indicated (112, 113). Only about 5% of lung cancer patients have the ALK gene fusion (113, 114), which means a great deal of screening was required to identify and enroll patients in the crizotinib studies. This was very inefficient compared to a “basket trial” design (115) in which patients are screened simultaneously for a large number of genetic aberrations using a multi-gene panel to determine their eligibility for a large number of clinical trials involving different therapeutic interventions. Some forward-looking models in this area propose a multi-institution collaboration that employs a multi-gene panel assay in which the cost of the screening assay (including validation) is shared by different drug development entities (49, 115, 116). While this approach would significantly reduce the cost of screening patients for rare subpopulations of patients in PhII and PhIII trials for each individual company, it presents the equally interesting question of whether drug developers will collaborate with competitors in such basket trials. The Friends of Cancer Research initiative for enrollment of patients with advanced NSCLC into trials matched by their tumor profile is one of the first examples of this kind of study (49). The trial seeks to utilize a NGS panel-based approach for enrolling patients into the most suitable trial using an adaptive trial design that allocates patients to suitable drugs from different pharmaceutical participants. It includes five drugs from five different companies and will employ the FM NGS-based panel assay to guide subject assignment and is expected to launch in spring 2014 (116). Overall, drug developers and Dx companies will have to work together to navigate this disorderly transition in testing paradigm (12).

### Drug development challenge 4 – when to commit to co-diagnostic development?

An important question arises as to when the pharmaceutical company should invest in the NGS co-Dx development process. The best guidance would dictate that CDx assay development must begin at least 18–24 months prior to the start of the registrational studies to allow sufficient time for development of the IUO assay for demonstration of clinical utility in the registrational trial. The Dx development plan depends on many factors such as complexity of the assay, cost of pre-investment, strength of the data confirming the biomarker hypothesis, as well as timeline of drug registration (e.g., whether a traditional Phase 2 to Phase 3 transition timeline is expected) (117). Therefore, variation to the ideal development timeline is often observed and drug companies and Dx developers utilize different developmental strategies to develop the final IVD product with significant investment by both parties (Figure 4). Development of a CDx test typically links the market uptake and return on investment of Dx device to the performance of the companion drug in pivotal clinical trials. As a consequence, the cost of development may require creative cost sharing and milestone payment agreements between the pharmaceutical and Dx partner. Some of the plausible developmental strategies possible for current NGS-based Dxs may be summarized as follows:

(i) Linear, risk-averse development model: in this model, development proceeds by a linear, logical flow, minimizing investment risk by delaying decisions as long as possible. CDx development is only begun after the need for a CDx is unequivocally established or until after initial data show the therapeutic has efficacy. Although avoiding pre-investment in Dx development until it is clearly needed may appear to be wise, in reality this may be a poor strategy because once it is clear that the drug is effective, there will be a great urgency to initiate the pivotal studies. The second aspect of risk aversion is the desire to avoid a bridging strategy for the Dx, i.e., starting the Ph3 studies with CTA instead of an IVD-ready version of the Dx (i.e., the IUO version of the assay) and then transitioning, i.e., bridging, to the IUO version by re-analyzing all (or nearly all) of the samples on the IUO version of the assay. This transition introduces significant risk into the process, so avoiding bridging is a good plan, but the cost is a significant delay in the start of the pivotal trial.
(ii) Pre-investment model: the Dx partnership is finalized and the IVD assay development starts prior to the initiation of the Phase 2 study, allowing sufficient time for development of the IUO assay to be completed prior to the Phase 3 start. In this case, the Dx development risk is low, but the Dx utility and therapeutic development risks are high. This is because the Dx development starts before the therapeutic is shown to be effective and before the Dx is shown to be required. Thus, the key risks are the uncertainty of biomarker’s clinical utility and the therapeutic’s clinical efficacy from Phase 2 data. Although the therapeutic sponsor partner may essentially partially fund Dx development as part of the Dx agreement, the therapeutic sponsor does not absorb all the risk. Dx companies have limited resources and have to select partnerships most likely to lead to a successful Dx product launch. In other words, one of the risks felt by the Dx company is opportunity cost if the program is canceled for any reason, including the failure of the therapeutic.
(iii) Bridging strategy + partial pre-investment. In cases where the traditional 18–24-month window for pivotal trial start is not possible, this model may be utilized to allow a pivotal trial start in a timely manner. This is a very expensive strategy with the drug sponsor absorbing most of the risk. IVD assay developments starts with a prototype assay (non-NGS or NGS-based) and bridging studies proceed as soon as an IUO version of NGS-based assay is ready. This strategy suffers from having high sample requirements as well as necessitating sample stability studies.

### Drug development challenge 5 – type of a CDx device: LDT or kit?

Many of the early leaders in precision medicine, realizing the possible complexity of the traditional PMA regulatory path for CDx kit development, may consider the LDT IVD model for their therapeutic that requires an *in vitro* Dx. This scenario could arise if the drug maker wants to avoid a large upfront investment in a CDx effort and has identified a reliable partner that can develop an acceptable assay, support clinical trials and provide worldwide access to the assay in their laboratory. The LDT route might also be selected if the company only recognizes it needs an IVD late in clinical development (i.e., in PhII) and wants to avoid a bridging strategy. Even though an LDT can receive FDA-clearance through the 510(k) process (118, 119), it seems likely that the FDA would require the LDT to go through the PMA process. Thus, the main advantage of the LDT route would be to avoid investment in a traditional kit and to avoid a delay related to the development of the IUO device. The current debate on stricter regulation of LDTs may play an important role in such decisions (75 FR 34463, 2010). Variability in LDT design and the increase in number of LDTs over 510(k)-cleared Dx devices is a growing concern (14), since it would take enormous efforts to standardize LDTs to achieve universally accepted tests. Standardization and strict regulation of CLIA NGS LDTs may be the practical scenario encountered most in next few years. As the FDA’s guidance and recommendations for LDT regulation become clear and start getting enforced, the clinical NGS field will see standardization at many diverse levels, e.g., controls used in assays, reagents/panels, assay QC parameters and rules for accepting or failing data, bioinformatics pipelines and bio-statistics modules, interpretation of data, reporting of data, etc. Key considerations must include early adoption of the Dx assay, preferably prior to pivotal studies. As discussed under time line constraints and in Figure 4, not many current NGS-based assays are suitable as Dxs or are ready to be developed into a regulated Dx.

### Drug development challenge 6 – how should clinical actionability be defined?

While detection of low frequency mutations is one of the great promises of a NGS-based Dx, detection of very low frequency mutations in a Dx test requires several serious design considerations as well. For example, even if a test is technically able to detect a very low frequency mutation (e.g., <1%), the presence of the mutation may not correlate with therapeutic response since the majority of the tumor (>99%) ultimately does not carry the said mutation. In this case, reporting of the detected mutation may require special consideration. For example, if the said mutation were present at 5% allele frequency, the Dx might report the mutation present and qualify the patient for treatment with the paired pharmaceutical, but if at 0.5%, it might not. In other words, a scenario is possible where patients with a low frequency mutation detected by a Dx test may be ineligible for a clinical trial due to mutation frequency actionability thresholds (41, 120). However, while not “pharmacologically” actionable, the 0.5% mutation detected would likely require reporting for follow-up. Ultimately clinical utility of low frequency mutations will be demonstrated by clinical response, which will provide clarity on what level of sensitivity of mutation detection is acceptable for drug labeling. Similarly, tumor heterogeneity may reveal mutations in a gene or transcriptional changes that are not yet clinically actionable.

### Drug development challenge 7 – what is the ex-US regulatory environment for NGS diagnostics?

In some situations, the Dx that supports approval of a drug outside of the US will be different than the assay that is approved by FDA. This can be due to a number of factors including the US testing being a lab-based assay or the readiness of the Dx company to support distribution worldwide. Additionally, it is particularly important that the policies governing genetic data collection, reporting, and analysis be clear from the start of a Dx program in a territory. In the EU for example, a CDx is not specifically formally classified, though the regulations may change soon (121). However, the test must be CE marked under the EU IVDs Directive (122, 123). The clinical trial use of the test can then be included in the label following a European Commission approval.

### Diagnostic development challenge 1 – additional regulatory guidance

Through the MiSeqDx decision summary, Dx companies are just now getting a glimpse into FDA thinking regarding NGS technology and the use of multi-gene panels. The FDA has indicated that a guidance on regulated NGS assays is due in 2014 and has proposed that individual companies request early pre-submission meetings with the Agency to discuss Dx development plans and trial design. It is encouraging that FDA officials have offered at public forums personal opinions that convey the Agency’s enthusiasm about the technology and its application for therapy, as well as the recognition of the inevitability of usage of NGS-based tests in public health (focr.org). The FDA has encouraged early and open dialog on the NGS CDx process and has implied that the process, in spite of its complexity, is likely to be facilitated in a manner as similar as possible to that done for existing complex Dx assays.

### Diagnostic development challenge 2 – competition from LDTs

The current environment is one in which NGS-based lab-developed tests are rapidly gaining popularity in the healthcare community and the growing use of NGS-based cancer genome profiling may be pushing the community toward a fast adoption of NGS-based tests. Although there are several sets of guidelines and recommendations (CLIA, CAP, and state guidances) (43, 44) describing the validation and use of existing NGS LDTs, the FDA has indicated that regulation may be necessary to stop the growth of less rigorously validated assays and to reduce the risk to patients. The oncology community’s clamor for an information rich NGS Dx is possibly similar to the initial excitement around using microarrays as Dxs, with the goal of having a single comprehensive test that captures a large amount of relevant content. Tests that identify patients that benefit, or not benefit, from certain treatments represent new opportunities and a new market for some companies. Many different types of companies are building research usage only (RUO) cancer panels in the expectation that they could be adopted as LDTs. Other companies are setting up laboratories or expanding their current laboratory capabilities to offer LDT cancer panels and other NGS-based tests (47, 124). The latter represent a significant threat for Dx companies and may make them hesitant to invest heavily in developing an FDA-approved Dx product, especially as less regulated LDTs continue to increase their segment of the Dx market. For example, the recently FDA-approved molecular Dx BRAF V600E test was followed by the development and rapid uptake of cheaper LDTs. FDA recently issued a guidance document (Distribution of IVD Products labeled for Research Use Only or IUO) which may address some of the issues with RUO marketing in particular.

### Diagnostic company challenge 3 – LDT versus IVD kit considerations

Both LDTs and kit-based Dxs are considered to be *in vitro* Dxs by the FDA, and either can go through the PMA process. Thus, one of the major decisions for NGS-based Dxs developers will be choosing between development of a LDT (currently working under enforcement discretion from FDA regulation) or a commercialized kit-based FDA-approved product. In this context drug companies can choose to partner with “traditional Dx companies” which do not work with a LDT model (they don’t have or want a CLIA service lab) or with “Lab-focused Dxs companies” which have a CLIA service lab and that could potentially offer an LDT-based Dx.

Currently, the NGS-based genetic tests on the market are all CLIA/CAP-regulated lab-developed tests (11). To date, none of these tests have been cleared or approved through FDA’s stringent pre-marketing review process, which verifies the performance claims of the test. To date only a very small number of molecular genetic tests have FDA approval for marketing as CDxs. Examples of FDA-approved kitted CDxs are the Roche COBAS 4800 test for BRAF V600E mutation detection as a CDx for vemurafenib (Zelboraf) and the Abbott Vysis ALK Break Apart FISH Probe test to identify ALK-positive NSCLC patients for Pfizer’s approved NSCLC therapy Xalkori (Crizotinib) (108, 109, 113, 125). There is a separate class of lab-based, FDA-cleared IVDs, e.g., the Agendia MammaPrint assay (126) and the XDx AlloMap assay (127). The largest class of genetic tests is currently unregulated clinical lab-developed tests. Clinical labs are overseen and regulated by the Centers for Medicare and Medicaid Services (CMS) with the CLIA certification process (40). Lab-developed test markets have grown mainly because the FDA approval process is time-consuming and very expensive (117). The extensive clinical validation and design control requirements expected in FDA-regulated IVD products are deterring many companies from submitting their NGS-based tests for the IVD process. At the same time, valid concerns about the lack of regulatory oversight that allows tremendous variability in test results from LDTs have led to a call for stricter regulation of the LDT (14). The FDA has sought more involvement in LDT regulation for a while now and there is increasing indication that LDT regulation will be on FDA’s agenda as evident in FDA’s Guidance on Personalized Medicine from October 2013. In 2010, FDA announced plans to expand its regulation to lab-developed genetic tests. This announcement led to heated debate within the industry (117). While this is yet to happen, it may impact the clinical LDT format of NGS assays should they become a specific CDx that requires FDA clearance or approval.

## Concluding Thoughts

We have provided a summary of the practical challenges to the widespread adoption of NGS-based CTAs and their further development as CDxs. For some challenges we suggested possible remedies that alleviate some of these concerns; for others we framed the relevant questions from a stakeholder’s perspective.

It is certain that despite the challenges, in the near future NGS-based Dxs will be a major component of the highly remunerative personalized medicine and Dx industry. What was said about genome sequencing may also be true for clinical NGS-based Dx testing: that we may be overestimating the impact in the short run but we are probably underestimating the impact in the long run (original quote is attributed to renowned futurist Roy Amara). It is a certainty that the healthcare system will be transformed if the technology is embraced and implemented into clinical practice with its full potential. We project that a variety of NGS Dx associated companies or specializations will see exponential growth as they aid the simplification of NGS in the clinic, especially those that offer easy-to-use clinical interpretation interfaces or EMR data incorporation methodologies. It is also foreseeable that clinical NGS will be coupled with methods for minimally invasive monitoring utilizing bio-fluid-based assays instead of traditional tissue biopsies. It is also notable that as pharmaceutical companies and healthcare systems drive clinical NGS into practice, several models for global collaboration between pharmaceutical companies may arise which can help the field of personalized medicine move forward exponentially.

## Conflict of Interest Statement

Applies to all authors: Merck purchases reagents and instruments from Illumina and Life Technologies and owns an Illumina MiSeq instrument and an Ion Torrent Personal Genome Machine. Merck has purchased services from Foundation Medicine using the Foundation One multi-gene NGS panel.

## Footnotes

^1^www.iontorrent.com

^2^www.illumina.com

## Abbreviations

ABRF, Association of Biomolecular Resource Facilities; ACMG, American College of Medical Genetics and Genomics; BRAF, v-Raf murine sarcoma viral oncogene homolog B; CAP, College of American Pathologists; CDER, Center for Drug Evaluation and Research; CDRH, Center for Devices and Radiological Health; CDx, companion diagnostic; CE Conformité Européenne, Conformity Marking for Relevant European Council Directives; CFTR, cystic fibrosis transmembrane conductance regulator; CLIA, Clinical Lab Improvement Amendment; CRO, contract research organization; CTA, clinical trial assay; ddNTPs, dideoxynucleotide triphosphates; DNA, deoxyribonucleic acid; dNTPs, deoxynucleotide triphosphates; Dx, diagnostic; EGFR, epidermal growth factor receptor gene; EMR, electronic medical records; ePCR, emulsion PCR; FDA, United States Food and Drug Administration; FFPE, formalin fixed paraffin embedded; gDNA, genomic DNA; IHC, immunohistochemistry; ISPs, ion sphere particles; IUO, investigational use only; IVD, *in vitro* diagnostic; KRAS, Kirsten rat sarcoma viral oncogene homolog gene; LDT, lab-developed test; MAQC, microarray quality control; NCI, National Cancer Institute; NGS, next-generation sequencing, NIST, National Institute of Standards and Technology; NTC, no template control; PacBio, Pacific Biosciences; PGM, Personal Genome Machine; PMA, pre-market approval; PT, proficiency testing; QC, quality control; qPCR, quantitative polymerase chain reaction; RNA, ribonucleic acid; RNA-Seq, RNA sequencing; RUO, research usage only; SMRT, single molecule real time sequencing; SNV, single nucleotide variant; TAT, turnaround time; UTR, untranslated region; VCF, variant calling file; ZMW, zero-mode waveguides.
